# Everybody's Page

**Published:** 1889-05-11

**Authors:** 


### Everybody's Page.

According to M. Moulé, domestic fowls are very subject to tuberculosis of the abdominal organs. *Paté de foie gras*, the epicure's delight, is often a pure culture of tubercle bacilli. This is an appetising thought.

In sunny Italy the Royal Family suffer from chest diseases. The King has never wholly got over the attack of congestion of the lungs to which he nearly succumbed a few ears ago, and the Crown Prince has a tendency to consumption.

Spirit of ammonia is the best thing to clean hair-brushes with, as it does not soften the bristles like soap or soda. If a teaspoonful of ammonia is mixed with a quart of water, the brush need only be dipped in the solution for a moment, and all grease is removed. The brush should then be rinsed in cold water, shaken well, and dried in the air, but not in the sun.

We have our little snobbish ways on this side of the water, but our Republican cousins can beat us in this as in many other things. At least we do not know of any English tradesman who has perpetrated an advertisement like the following triumph of an American perfumery company: "Orchids are the most aristocratic of flowers, and Orchid Perfumes the Royal Family in the world of fashionable odours."

The *Bristol Medico-Chirurgical Journal*, in noting the publication of a new book on the diseases of women, says: "Whatever just cause of complaint women may have as to their being overlooked in suffrage, science, art, or matrimony, they certainly cannot complain that they are neglected as to their diseases." Alas, poor women! Sometimes one inclines to think that in this matter "a little wholesome neglect" would do no harm.

Sanitary inspectors are not confined to the human race. The jackdaw has appointed himself to a similar and very useful office with regard to sheep. Perhaps his action is not wholly unselfish, but the sheep are none the less grateful. Jack, flying overhead, selects a likely animal, settles on his back, and proceeds to free him from his insect tormentors. He goes through his work steadily and conscientiously in a business-like fashion; while the sheep stands still, conscious, it would seem, that the bird's invasion is all for his good. If the sheep moves with the rest of the flock the jackdaw moves with it, indifferent to everything but the work on hand. Only when he has satisfactorily finished his self-imposed task does he go away, and then it is to perform a similar service to some other member of the flock. The same service is performed for the sheep by the starling also.

The *Sanitary News* is an advocate of vegetarianism, and quotes in support of its views an account of a certain tribe in Bengal, the Oswals of Marwac, who are forbidden by their religion to eat the flesh of any animal or to drink any alcoholic liquor, and among whom cholera is unknown, although it has several times appeared among neighbouring tribes. On the other hand, Dr. Hendley, of the Mayo Hospital at Jeypore, among whose native patients are many pure vegetarians, finds the avoidance of animal food no barrier to the inroads of many diseases. Cancer, from which M. Reches declared vegetarians to be free, does not seem to spare them at Jeypore. At least, of 102 operations for cancer which Dr. Hendley has performed since 1880, 61, more than half, have been on vegetarians, and six of these were of the strictest sect of Jains, who do not consume even many vegetables, but confine their diet to fruit and milk. We fear the vegetarians must content themselves with modifying and supplementing our present fashion in food. It is not likely that they will ever supplant it.

Silk was first introduced into Europe in 552, when some Greek monks, who had wandered so far as China, brought some mulberry seeds and silkworms' eggs to the Emperor Justinian at Constantinople, hidden in the hollow of a cane.

"You think," said the coroner, "that the body on which the inquest is being held is that of your friend, who disappeared from home three weeks ago?" "Yes," said the witness. "Then," the coroner asked, "is there any characteristic by which you could identify him?" "Yes, easily," was the reply. "He stuttered horribly."

He had two wooden legs, but as it was about a cold in the head he was consulting his doctor, it did not seem necessary to mention the fact, and when he was told to go home and bathe his feet in hot water, he religiously did as he was told. But he complained afterwards that the treatment hadn't done him a bit of good. Strange!

We all know, some of us by bitter experience, one use of castor oil, but there are many others. It is the best foundation for hair oil; it is used in preparing textile fabrics for dyeing; it is admirable for dressing tanned hides; it is an excellent lamp oil, superior to petroleum, and from it a very good gas can be distilled; it is very well adapted for lubricating fine machinery; and finally, when dissolved in alcohol, it makes a good varnish.

Two rather peculiar cases of lead poisoning were lately reported to the Practitioner's Society of New York. The victim in the first was a florist, who had been in the habit of biting off the ends of the tin-foil used as wrappers for bouquets. This tin-foil contained about eighty per cent. of lead. In the second case the poisoning had been induced by drinking beer out of bottles that had been cleaned with lead shot.

What are we to burn when the coal mines are exhausted? The question has often been raised, and never, so far as we know, satisfactorily answered, the general feeling on the subject being pretty much, "Let posterity look after itself. Posterity hasn't done anything for us." But in California the question is being answered. It is found that peach stones burn as well as the best coal, and give out more heat in proportion to weight. At the present time the stones taken out of the fruit that is tinned or dried are collected, and sell at the rate of 24s. a ton. Apricot stones also burn, but not so well as peach, and do not command so good a price. But it is sad for a fruit lover to reflect that this fuel will never be common in England.

Boerhave, the famous physician, declared that a man was more likely to get well by climbing a tree than by drinking a decoction made of its leaves; that is, he thought exercise better than medicine. It is on this principle that the Queen of Sweden, whose nervous condition has given rise to much anxiety, is being treated. She is ordered to make her bed and sweep her room, besides taking a large amount of walking exercise. This method—the "housemaid treatment," as he calls it—has inspired a cynical journalist with some suggestions which are, perhaps, wiser than he knows. He advises the "office-boy treatment" for the dyspeptic millionaire, the "groom treatment" for the Crœsus whose liver is too much with him, the "country postmen treatment" for the obese financier; the "nursemaid treatment" for the hysterical woman who cannot stand a child's cry, and the "old-clothes woman treatment" for the fine lady who faints at the sight of a powder. Probably the "treatments" would be efficacious—if the patient would submit.

